# Can ecosystem-scale translocations mitigate the impact of climate change on terrestrial biodiversity? Promises, pitfalls, and possibilities: Ecosystem-scale translocations

**DOI:** 10.12688/f1000research.7914.1

**Published:** 2016-02-08

**Authors:** Stéphane Boyer, Bradley S. Case, Marie-Caroline Lefort, Benjamin R. Waterhouse, Stephen D. Wratten

**Affiliations:** 1 Department of Natural Sciences, Faculty of Social and Health Sciences, Unitec Institute of Technology, Auckland, New Zealand; 2 The Bio-Protection Research Centre, Lincoln University, Lincoln, New Zealand; 3 Department of Informatics and Enabling Technologies, Faculty of Environment, Society and Design, Lincoln University, Lincoln, New Zealand

**Keywords:** Climate change, Translocation, Conservation Biology, Ecological Restoration, Terrestrial Ecosystems, Species Interactions

## Abstract

Because ecological interactions are the first components of the ecosystem to be impacted by climate change, future forms of threatened-species and ecosystem management should aim at conserving complete, functioning communities rather than single charismatic species. A possible way forward is the deployment of ecosystem-scale translocation (EST), where above- and below-ground elements of a functioning terrestrial ecosystem (including vegetation and topsoil) are carefully collected and moved together. Small-scale attempts at such practice have been made for the purpose of ecological restoration. By moving larger subsets of functioning ecosystems from climatically unstable regions to more stable ones, EST could provide a practical means to conserve mature and complex ecosystems threatened by climate change. However, there are a number of challenges associated with EST in the context of climate change mitigation, in particular the choice of donor and receptor sites. With the aim of fostering discussion and debate about the EST concept, we  1) outline the possible promises and pitfalls of EST in mitigating the impact of climate change on terrestrial biodiversity and 2) use a GIS-based approach to illustrate how  potential source and receptor sites, where EST could be trialed and evaluated globally, could be identified.

## Introduction

Populations of animals and plants facing insurmountable barriers to dispersal, and species with low dispersal abilities are likely to be highly impacted by climate change ^1^. With temperatures changing in their historical distribution area, such species may not be able to colonise new habitats with more hospitable climatic conditions and may require human intervention in the form of assisted colonisation if they are to survive ^2, 3^.

The translocation of endangered species has been used as a conservation management tool for several decades ^4, 5^ and assisted colonisation as defined by Ricciardi & Simberloff (2009) appears as a logical tool for mitigating the impact of climate change on terrestrial organisms ^7^. However, conservation translocations can be logistically difficult and costly ^8^ and can often fail ^7, 9, 10^. One potential cause of failure is that the complex community interactions of which the translocated species are a part are left behind. Such interactions are likely to be required for long-term survival of the target species but may not be present or fully functioning at the appropriate rate at the translocation site ^11^. These interactions are also likely to be the first component of the ecosystem to be impacted by climate change ^12^, long before any population or species goes extinct. Conservation strategies should therefore have a greater focus on the translocation of whole ecosystems and their inherent interactions rather than that of individual ‘flagship’ species ^13^. This paper explores a novel approach – ecosystem-scale translocation (EST) – as a means to preserve functioning terrestrial ecosystems threatened by climate change. This involves the careful collection and immediate transfer of topsoil, vegetation and associated ecological communities to a receptor site. Ecosystem-scale translocation may represent an immediately available and practical method for preserving communities and ecosystems threatened by climate change. Here, with the aim of fostering discussion and debate about the EST concept, we 1) outline the possible promises and pitfalls of EST in mitigating the impact of climate change on terrestrial biodiversity and 2) use a GIS-based approach to illustrate how potential source and receptor sites, where EST could be trialed and evaluated globally, could be identified.

### Rationale for the proposed approach

Species interactions are essential to the functioning of ecosystems. However, in the context of climate change and biodiversity crises, understanding the full range of community interactions that are needed to recreate a functioning ecosystem in a potential receptor area is not realistic ^14^. It follows that ecological engineering through manual planting or regeneration through the seedbank, as is applied in classical restoration programmes ^15^, is not a practical solution for conserving functioning above- and below-ground communities of ecosystems threatened by climate change. On the contrary, the translocation of topsoil, vegetation and all the communities they contain could represent a much-needed shortcut where all components and interactions of an ecosystem are potentially preserved.

### Existing examples of ecosystem translocation

**Figure 1.**
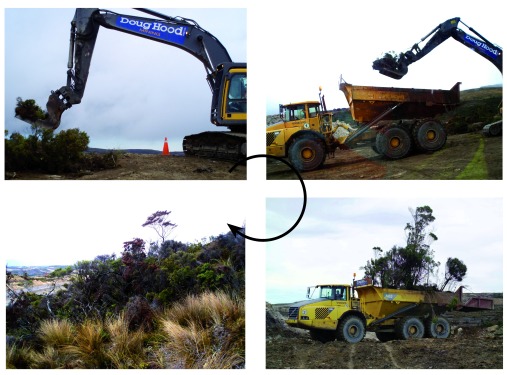
Vegetation direct transfer of native alpine forest at the Stockton opencast coal mine (New Zealand). Clockwise: pieces of land comprising vegetation, roots and soil are cut; soil and vegetation sods are loaded on a truck and transported to the receptor site; the landscape jigsaw is then reconstituted with minimum gaps between the sods to recreate a continuous habitat. Credits: Stéphane Boyer and Solid Energy New Zealand Ltd.

Under the terms *habitat translocation*, *community translocation*, *vegetation translocation* or *transplanting,* ecosystem translocation has been applied at a small scale to conserve particular plant species or communities impacted by proposed land development ^16^ but also to test the robustness of plant communities in climatically challenging conditions ^11, 17^. A number of earlier translocation attempts reviewed by Good *et al.* ^18^ showed that with appropriate preparation of the receiver sites, translocation of turves produced satisfactory results in terms of plant survival and conservation of community composition for a variety of plant community types. In the mining industry, a similar process called *vegetation direct transfer* (VDT) has been applied for 30 years as part of mine restoration programmes ^19– 21^ sometimes translocating large areas. For example, a total of 75 ha of native grassland, shrubland and low canopy forest have been translocated within the Stockton Mine (New Zealand) in the past 30 years ^22– 24^. The method used consists of (i) cutting pieces of land comprising soil, roots and vegetation over a 1–3 m ^2^ area and a depth of 30 cm or more using a digger, (ii) transporting these sods by truck to a new area and (iii) reconstituting the ‘jigsaw’ with minimum gaps between the sods to recreate a continuous habitat ( Figure 1). Many studies have highlighted the positive outcomes of such translocations, including: the conservation of plant and beetle communities, soil functions and microbial activity after wetland translocation ^25, 26^; the maintenance of soil structure and fertility in translocated grassland; rtebrates (e.g. carabid beetles, weta) and plant species (beech) from shrubland and tussock wetlands ^22^; the preservation of biodiversity and biomass in soil fauna ^27^ and microbial communities ^28^ from alpine vegetation; and the conservation of habitat for birds and invertebrates from translocated forest areas ^21^. There is also an extensive body of work, including peer-reviewed articles ^21, 24, 26, 29^ and reviews ^18, 19, 21^, but also internal reports and conference presentations (see Supplementary material), most of which point to the conclusion that VDT promotes faster ecosystem recovery than do other conventional restoration techniques such as replanting or hydroseeding.

### Ecosystem-scale translocation - promises

**Figure 2.**
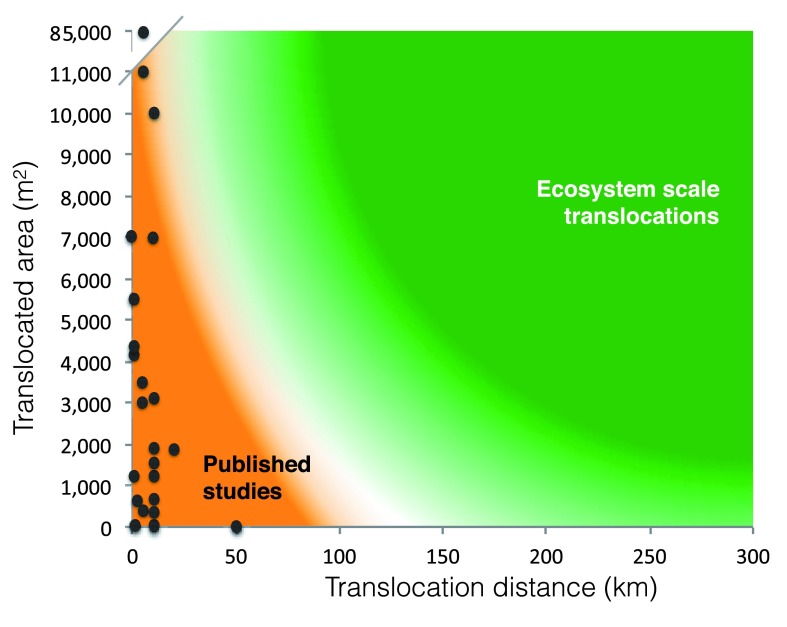
Conceptual diagram of the size of the area translocated in relation to translocation distances. The orange area corresponds to typical past and current habitat translocation projects, with dots corresponding to published studies (see Supplementary material S1 for a complete list). The green area represents an ideal combination of size and distance for ecosystem-scale translocation, with darker green more suitable, although more difficult to put in place.

Could ecosystem-scale translocation represent a practical and economic translocation method, by which all or most immobile, slow moving and low-dispersal elements of a terrestrial ecosystem are moved together as a functioning community of above- and below-ground organisms? While no taxonomic group is particularly targeted by EST, it is likely that plant, microbial and fungal communities as well as most invertebrates and small vertebrates will directly benefit, while large and mobile terrestrial vertebrates will probably vacate the site due to disturbance during the translocation process. Therefore, EST constitutes a complementary approach to single-species conservation translocation programs, which suffer from a significant taxonomic bias towards larger animals. Indeed, almost 60% of all animal translocation efforts to date have been for large and charismatic species of birds and mammals ^7^, despite these taxa comprising only c. 1% of all known animal species ^30^. Although translocation distances and the sizes of the areas translocated are usually minimised for economic and practical reasons in a restoration context ( Figure 2), this process could be scaled up for the purpose of ESTs where the aim is to mitigate the impact of climate change on biodiversity in highly-valued ecosystems. The aim of EST should be to preserve a functioning representative subset of an ecosystem of sufficient size to ensure its long-term survival and functioning. Capturing most ecosystem functions is likely to require the translocation, in stages, of fairly large areas. However, the majority of the current protected areas globally are less than 1,000 ha and places such as Barro Colorado in Panama (1,500 ha), the Bosavi Crater in Papua New Guinea (1,250 ha), or Darwin Island in the Galapagos (110 ha), are good examples of relatively small areas with significant biodiversity interest.

### Ecosystem-scale translocation - pitfalls

Only a handful of translocation studies have been reported in the scientific literature ( Supplementary material) and the extent to which biodiversity conservation was measured in these studies is very limited. More targeted research is therefore required to assess to what extent EST can conserve biodiversity at a range of levels including species, genetic and functional diversity, as well as species interactions and ecosystem functions. Critical factors and recommendations required for habitat translocation include similarity between the environmental conditions of the donor and receptor sites, the translocation technique, and appropriate management of the translocated habitat ^31^. In particular, the nature of the bedrock strata as well as the geochemistry, hydrology and precipitations at the receptor site will have a strong influence on the translocated topsoil. Geology can be measured and matched between donor and receptor sites, while the range of possible changes in fine-scale hydro-dynamics may be much more difficult to predict as climate changes ^32^. One additional and outstanding issue is the selection of a candidate receptor site where the ‘graft’ will not replace communities which are themselves of high-value, possibly leading to the extinction of local species ^14^ or negatively impacting the functioning of surrounding ecosystems ^6, 9^. For example, some phytophagous insects could rapidly become invasive when encountering new plant species within their distribution range ^33^. Conversely, the spillover of existing invasive species from areas surrounding the receptor site could negatively impact the graft itself. This is possibly of less concern because species invasion is less likely to happen when translocating complete and functioning ecosystems where most or all niches are occupied and, consequently, where the lack of empty niches make ecosystems more resistant to the establishment of invasive species ^34^. Supposedly, invasion risk could also be reduced if the receptor site is located, within areas where species composition does not differ dramatically from that of the donor site. Nevertheless, it is important to note that any negative impacts are likely to be difficult and costly to reverse after translocation and a thorough risk assessment would be required to ensure that translocated species do not affect the surrounding areas after translocation.

The cost involved in the translocation of ecosystems is likely to constitute an important limitation. Because only few examples exist, it is difficult to provide a definite price per unit area. One comparative study by Simcock *et al.* ^22^ showed that translocation in a mine rehabilitation context was seven times more costly than transporting bulldozed soil and vegetation, but twice cheaper than rehabilitation through nursery cultivation followed by manual replanting. This study was based on the translocation of a 0.6 ha of native forest over a few hundred meters distance and most of the additional cost was from transportation between donor and receptor area as trucks could only carry a single layer of sods (i.e. 30 to 50% of full capacity) ^22^. Translocation of larger areas over much longer distances, as required for EST ( Figure 2), is likely to be much more costly.

### Potential receptor sites

In addition to a deep understanding of the risks and potential side effects, the application of EST also requires carefully selected receptor sites. Suitable receptor sites should have very low intrinsic biodiversity value because they will be replaced by EST and whatever communities were present will disappear. Such potential sites could be selected in agricultural land that has been marginalised or abandoned due to the erosion of the fertile soil layer and the loss of essential soil nutrients following overexploitation or misuse ^35^. It has recently been estimated that 25% of all terrestrial habitat is highly degraded ^36^, with one hectare of productive land lost globally every 7.67 seconds ( www.irri.org). There is growing interest in using such marginal and degraded land to cultivate biofuel crops ^37, 38^. However, this land could alternatively be used for biological conservation. When soil is lost or degraded to the extent where growing crops is not possible, the land could still be suitable for receiving EST, because the latter includes topsoil and its associated ecosystem functions. With human-degraded soils scattered across all continents ^37^ potential receptor sites could be available in a range of climate zones.

Assisted colonisation of single species includes moving organisms to habitats with more favorable future climatic conditions ^5^. The timing of assisted colonisation is therefore crucial because those performed before the recipient region is predicted to be climatically suitable are likely to fail ^39^. Determining the “window of opportunity” – a scenario where an ecosystem is still viable at its original location, and where there is an existing suitable receptor site - is likely to prove difficult and will require active adaptive management ^40^. To alleviate the above limitation, EST could be applied to move ecosystems from climatically unstable areas to areas where the climate is projected to remain stable despite global changes ^41^. The climate stability index proposed by Iwamura *et al.* ^42^ can be used in this context. This index compares observed temperature, precipitation, cloud cover and vapour pressure in 1997–2002 to projected values in 2047–2052. Human-degraded soils that occur in climatically stable areas could offer prime locations to receive ESTs and could receive them from regions of high conservation value (e.g. biodiversity hotspots) currently located in unstable climatic areas.

## Results

### An illustration of the potential for EST

Using a highly conservative GIS approach (see Method section), we identified a range of potential source and receptor areas at a global scale. The former were located mainly in North America and North Eurasia as well as over the Amazonian forest ( Figure 3A), while a large majority of potential receptor areas were located in Africa, with smaller areas also identified in America, the Middle East, Asia and Australasia ( Figure 3B). The suitability of these source and receptor areas is largely dependent on the accuracy of current climatic predictive models, which are constantly improving ^43^. Also, climatic stability thresholds and criteria used for selecting highly valuable ecosystems or degraded land can vary greatly. In a similar way to conservation programmes, which often struggle to cross borders ^44^ due to different policy in neighbouring countries and a lack of global legislation, EST is highly unlikely to be applied in a cross-border situation (where an ecosystem with high conservation value would be translocated from one country to another). Therefore, we identified countries where large areas of both source and receptor sites were present ( Figure 3C). They include Madagascar, USA (California), Mexico, Turkey, Nigeria, Cameroon, South Africa, Vietnam, Thailand, Myanmar and Indonesia ( Table 1). These countries may therefore be ideal candidates to trial EST.

**Figure 3A.**
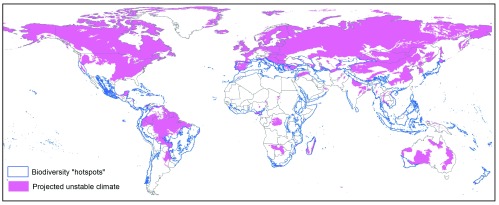
Global map of highly valued areas that could be considered as potential source for ecosystem-scale translocation. Ideal source areas are those where high value (blue lines) overlaps with low climatic stability (in pink).

**Figure 3B.**
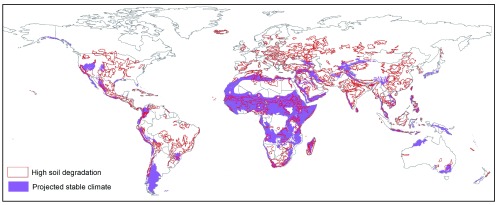
Global map of potential candidate receptor areas for ecosystem-scale translocation. Ideal sites are those where high soil degradation (red lines) overlaps with high climatic stability (in purple).

**Figure 3C.**
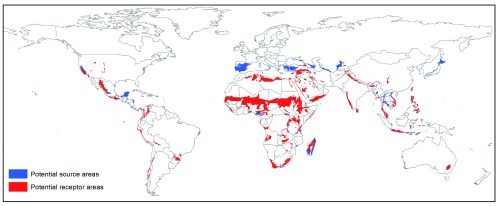
Global map of potential source (in blue) and receptor areas (in red) as defined in the legend of Figure 3A & Figure 3B.

**Table 1.** Surface of and distances between source and receptor areas within countries where both types of areas are present. Countries were ranked from best to worst candidates based on size of source area, size of receptor area and minimum distance between the two. Orange background: best candidate countries based on thresholds of 15,000 km ^2^ for the areas and 500 km distance between areas. Numbers in bold correspond to limiting factors. Blue background: countries where distance between source and receptor may be limiting using the above criteria; Purple background: countries where receptor areas may be limiting; Green background: countries where receptor areas may be limiting; Grey background: countries where multiple limiting factors exist. Min. source-receptor distance is the minimum distance between a patch of source area and a patch of receptor area. When source and receptor are adjacent, the minimum source-receptor distance is 0.

	Source	Receptor	Minimum source- receptor distance (km)
COUNTRY	Area (km ^2^ × 1000)	Area (km ^2^ × 1000)	
Turkey	225.564	99.797	0
Mexico	192.261	330.998	0
Madagascar	191.450	199.523	0
Thailand	81.181	54.266	0
United States	56.858	124.758	0
Nigeria	50.606	372.483	0
Myanmar	35.007	41.385	12
Cameroon	33.907	133.513	0
Vietnam	33.394	84.512	0
Iran	29.814	56.357	212
Indonesia	26.200	193.764	59
Australia	25.241	77.190	**895**
Brazil	17.338	110.477	**2067**
Cambodia	31.448	**13.063**	0
Kazakhstan	47.915	**6.220**	0
Mozambique	19.228	**3.757**	125
Uzbekistan	71.783	**1.390**	48
Azerbaijan	50.529	**0.807**	0
Russian Federation	22.149	**0.313**	111
South Africa	**12.459**	272.566	0
Malaysia	**7.924**	59.212	3
Colombia	**6.007**	151.639	22
Yemen	**3.722**	174.707	388
Eritrea	**2.848**	76.981	232
Philippines	**0.236**	168.094	38
Cuba	**11.976**	**1.735**	0
Laos	**11.564**	**0.620**	0
Dominican Republic	**8.754**	**0.211**	73
Puerto Rico	**8.641**	**0.094**	0
Morocco	**6.328**	**2.043**	34
Panama	**6.098**	**8.189**	2
Tajikistan	**3.877**	**4.157**	89
Haiti	**3.431**	**0.293**	3
Guatemala	**2.633**	**2.067**	70
El Salvador	**0.642**	**0.063**	33
Kyrgyzstan	**0.394**	**0.128**	158
Georgia	**13.127**	**3.720**	0
Chile	**0.045**	61.907	**628**
Ecuador	**7.813**	**8.460**	**927**

## Discussion

Clear evidence that ecosystems have started to shift in response to climate change is now observable, with plant ^45^ and animal ^46^ advancing towards higher altitudes and latitudes. Using the EST approach, it may be possible to move ecosystems as a preemptive measure to ensure that all immobile, slow moving and low-dispersal elements of a terrestrial ecosystem are moved together as a functioning community of above- and below-ground organisms.

Translocating communities and ecosystems should not be regarded as a substitute for less invasive conservation efforts when such options are available ^19, 47^, but considered only as a last resort, when ecosystems are at inevitable risk of disappearance or collapse. In the review by Bullock (1998), community translocation often did not achieve the preservation of complete and unchanged community, but led to communities ‘which resembles the pre-translocated state in mitigation for the loss of the original community and which retains many of the species found at the donor site. This method should therefore be recommended only when the aim is to mitigate inevitable negative impact on high-valued ecosystems. Important considerations associated with the proposed approach include the differences in bedrock strata, geochemistry and hydro-dynamics between the source and receptor site, the size of the translocated ecosystem subset in relation to edge effects and isolation, and potential negative impacts on the receptor site. These limitations will require careful planning on a case-by-case basis to minimise the risks of failure. However, in the face of the urgency of climate change, there is a pressing need for proposing and testing more proactive and ambitious forms of conservation management. Hoping that most species threatened by climate change will be able to (i) use environmental buffering ^48^; (ii) retreat to refugia ^49^; (iii) follow their climatic niche, potentially with the help of ‘corridors’ ^50^; or (iv) exhibit some form of phenotypic plasticity or micro-evolution on very short time-scales ^51^ is unlikely to be sufficient to conserve biodiversity at all scales. Ecosystem-scale translocation adds to the current debate on novel and audacious ideas to stem biodiversity loss, which include de-extinction ^52^ and the setting aside of half the planet for wildlife as proposed by E.O Wilson ^53^. Our analysis provides a starting point for discussion about the potential of EST for the conservation of ecosystems threatened by climate change. Although this is undoubtedly a radical option, and one that should be examined with caution, it could be applied to conserve very highly-valued ecosystems. Even if a number of species or functions were to be lost during the translocation process, this approach would still improve on the current limited single-species conservation translocation strategies. The EST approach is particularly suited to the conservation of small life forms, thereby balancing the current bias toward translocation and conservation of birds and mammals ^7^ and addressing the urgent issue of conserving invaluable invertebrate communities ^54^. This approach has the potential to add a new dimension to the spectrum of conservation tools currently available, with the aim of conserving mature and complex terrestrial ecosystems threatened by climate change.

Research is urgently required to assess to what extent EST can conserve not only species diversity, but also genetic diversity, functional diversity, species interactions and ecosystem functions. Source and receptor sites that ranked highly in this study would provide ideal candidates to test these hypotheses in medium to large-scale experiments of EST. This information will be critical to inform future EST projects, which are likely to become necessary if we are to tackle one of the biggest ecological challenges of the coming decades: preserving biodiversity at the ecosystem scale in an era of unprecedented climate change.

## Method

Literature included in Figure 2 was selected using the keywords ‘vegetation direct transfer’, ‘habitat translocation’, ‘vegetation translocation’, ‘community translocation’ and ‘vegetation transplant’ in all databases available in ISI Web of Science. This search was restricted to English written articles published from 1910 to 2016 (search conducted in January 2016). The reference lists of papers retrieved using these searches were then screened based on their titles for potential additional references. We used only those articles where the size of area translocated was reported and translocation distance was either given or could be estimated based on maps or GPS coordinates. Additional publications were extracted from the database of Solid Energy New Zealand Ltd. ( www.solidenergy.co.nz) and Landcare Research ( http://www.landcareresearch.co.nz) using the same search terms. All information used to build Figure 2 is summarised with the corresponding references in the Supplementary material.

We mapped the location of potential areas where EST may be applicable on a global scale by overlaying the GIS maps described below using ArcGIS 10.1. Potential source areas for EST were identified in GIS as those predicted to have a relatively unstable future climate based on global climate models, while also having high global conservation value. Climatically-unstable areas were identified using an ecoregion-based climate stability index dataset ^41, 55^, which incorporates seven different future climatic scenarios, and indicates how different future climate will be compared to present climate. This index ranges from 0.0 to 1.0 where 1.0 corresponds to complete overlap between current and future climates while 0.0 indicates no overlap ^55^. We averaged the climate stability indices across these seven climate scenarios; relatively climatically-unstable ecoregions were identified as those that had a mean stability index of <0.33 ( Figure 3A – pink areas). Areas of high global conservation values were identified using the Conservation International Biodiversity Hotspots dataset ^56^ ( Figure 3A – blue outlined areas). Potential receptor sites for EST were delineated in the GIS as areas that have both relatively high predicted future climate stability and a high incidence of soil degradation. Climatically stable areas were identified as ecoregions with a mean stability index of >0.66 ( Figure 3B – purple areas). Highly-degraded areas ( Figure 3B – red outlined areas) were extracted from a global soil degradation GIS dataset (GLASOD ^57^) as those polygons comprising degradation categories 3 and 4. The final composite map ( Figure 3C) shows the position of potential source and receptor sites together overlaid with country boundaries. For each country containing both source and receptor sites, we then calculated the total area of, and minimum distances between source and receptor sites within each country, allowing us to rank their potential as candidates for EST ( Table 1). We considered countries with source and receptor areas greater than 15,000 km ^2^ as good candidates because they offer a greater range in the choice of possible source and receptor sites. The other characteristic taken into account was the minimum distance between source and receptor areas. When this distance was less than 500 km, countries were ranked highly because road transportation is more likely to be completed within one day, therefore minimising the impact on the integrity of the transported vegetation and soil.

## References

[1] Thomas CD Cameron A Green RE: Extinction risk from climate change. *Nature.* 2004;427(6970):145–8. 10.1038/nature02121 14712274

[2] Fordham DA Watts MJ Delean S: Managed relocation as an adaptation strategy for mitigating climate change threats to the persistence of an endangered lizard. *Glob Chang Biol.* 2012;18(9):2743–55. 10.1111/j.1365-2486.2012.02742.x 24501053

[3] Müller H Eriksson O: A pragmatic and utilitarian view of species translocation as a tool in conservation biology. *Biodivers Conserv.* 2013;22(8):1837–41. 10.1007/s10531-013-0504-6

[4] Seddon PJ Armstrong DP Maloney RF: Developing the science of reintroduction biology. *Conserv Biol.* 2007;21(2):303–12. 10.1111/j.1523-1739.2006.00627.x 17391180

[5] Chauvenet AL Ewen JG Armstrong DP: Maximizing the success of assisted colonizations. *Anim Conserv.* 2013;16(2):161–9. 10.1111/j.1469-1795.2012.00589.x

[6] Ricciardi A Simberloff D: Assisted colonization is not a viable conservation strategy. *Trends Ecol Evol.* 2009;24(5):248–53. 10.1016/j.tree.2008.12.006 19324453

[7] Seddon PJ Griffiths CJ Soorae PS: Reversing defaunation: restoring species in a changing world. *Science.* 2014;345(6195):406–12. 10.1126/science.1251818 25061203

[8] Fonturbel FE Simonetti JA: Translocations and human-carnivore conflicts: problem solving or problem creating? *Wildlife Biol.* 2011;17(2):217–24. 10.2981/10-091

[9] Schwartz MW Martin TG: Translocation of imperiled species under changing climates. *Ann N Y Acad Sci.* 2013;1286:15–28. 10.1111/nyas.12050 23574620

[10] Sullivan BK Nowak EM Kwiatkowski MA: Problems with mitigation translocation of herpetofauna. *Conserv Biol.* 2015;29(1):12–8. 10.1111/cobi.12336 25040040

[11] Bruelheide H: Translocation of a montane meadow to simulate the potential impact of climate change. *Appl Veg Sci.* 2003;6(1):23–34. 10.1111/j.1654-109X.2003.tb00561.x

[12] Tylianakis JM Didham RK Bascompte J: Global change and species interactions in terrestrial ecosystems. *Ecol Lett.* 2008;11(12):1351–63. 10.1111/j.1461-0248.2008.01250.x 19062363

[13] Boyer S: Fauna in decline: the community way. *Science.* 2014;346(6211):821. 10.1126/science.346.6211.821-a 25395529

[14] Thomas CD: Translocation of species, climate change, and the end of trying to recreate past ecological communities. *Trends Ecol Evol.* 2011;26(5):216–21. 10.1016/j.tree.2011.02.006 21411178

[15] Bossuyt B Honnay O: Can the seed bank be used for ecological restoration? An overview of seed bank characteristics in European communities. *J Veg Sci.* 2008;19(6):875–84. 10.3170/2008-8-18462

[16] Trueman I Mitchell D Besenyei L: The effects of turf translocation and other environmental variables on the vegetation of a large species-rich mesotrophic grassland. *Ecol Eng.* 2007;31(2):79–91. 10.1016/j.ecoleng.2007.05.003

[17] Zhang F Li Y Cao G: Response of alpine plant community to simulated climate change: two-year results of reciprocal translocation experiment (Tibetan Plateau). *Pol J Ecol.* 2011;59(4):741–51. Reference Source

[18] Good JE Wallace HL Stevens PA: Translocation of herb-rich grassland from a site in Wales prior to opencast coal extraction. *Restor Ecol.* 1999;7(4):336–47. 10.1046/j.1526-100X.1999.72028.x

[19] Bullock JM: Community translocation in Britain: setting objectives and measuring consequences. *Biol Conserv.* 1998;84(3):199–214. 10.1016/S0006-3207(97)00140-7

[20] Cullen WD Wheater CP: Relocation and restoration in limestone quarries: implications for invertebrate communities following two extreme forms of management. *Proceedings of International Land reclamation and mine drainage conference and third international conference on the abatement of acidic Volume 3 of 4: Reclamation and revegetation drainage* Pittsburgh: United States Department of the Interior Bureau of Mines Special Publication SP 06C-94.1994;83–92. Reference Source

[21] Ross C Simcock R Williams P: Salvage and direct transfer for accelerating restoration of native ecosystems on mine sites in New Zealand. *New Zealand Minerals & Mining Conference Proceedings* 2000;97–104. Reference Source

[22] Simcock R Toft R Ross C: A Case Study of the Cost and Effectiveness of a New Technology for Accelerating Rehabilitation of Native Ecosystems. *Proceedings of the 1999 AusIMM Annual Conference* Canberra,2000.

[23] Simcock R Ross C: Guidelines for mine rehabilitation in Westland. Landcare Research - Manaaki Whenua,2014 Reference Source

[24] Rodgers D Bartlett R Simcock R: Benefits of Vegetation Direct Transfer as an Innovative Mine Rehabilitation Tool. In: Nichols O, Vikuckis N, editors. *Proceedings of the 2011 Workshop on Australian Mine Rehabilitation* JKTech Pty Ltd.2011;285–303. Reference Source

[25] Watts CH Clarkson BR Didham RK: Rapid beetle community convergence following experimental habitat restoration in a mined peat bog. *Biol Conserv.* 2008;141(2):568–79. 10.1016/j.biocon.2007.12.008

[26] Box J Brown M Coppin N: Experimental wet heath translocation in Dorset, England. *Ecol Eng.* 2011;37(2):158–71. 10.1016/j.ecoleng.2010.08.006

[27] Boyer S Wratten S Pizey M: Impact of soil stockpiling and mining rehabilitation on earthworm communities. *Pedobiologia (Jena).* 2011;54S:S99–102. 10.1016/j.pedobi.2011.09.006

[28] Waterhouse BR Adair KL Boyer S: Advanced mine restoration protocols facilitate early recovery of soil microbial biomass, activity and functional diversity. *Basic Appl Ecol.* 2014;15(7):599–606. 10.1016/j.baae.2014.09.001

[29] Box J Stanhope K: Translocating wildlife habitats: a guide for civil engineers. *Proc ICE-Civil Eng.* 2010;163(3):123–130. 10.1680/cien.2010.163.3.123

[30] The World Conservation Union: IUCN Red List of Threatened Species. Summary Statistics for Globally Threatened Species. Table 1: Numbers of threatened species by major groups of organisms (1996–2010).2010 Reference Source

[31] Box J: Critical Factors and Evaluation Criteria for Habitat Translocation. *J Environ Plan Manag.* 2003;46(6):839–56. 10.1080/0964056032000157624

[32] Allen MR Ingram WJ: Constraints on future changes in climate and the hydrologic cycle. *Nature.* 2002;419(6903):224–32. 10.1038/nature01092 12226677

[33] Lefort MC Boyer S De Romans S: Invasion success of a scarab beetle within its native range: host range expansion versus host-shift. *PeerJ.* 2014;2:e262. 10.7717/peerj.262 24795845 PMC3940619

[34] Levine J D’Antonio C: Elton revisited: a review of evidence linking diversity and invasibility. *Oikos.* 1999;87(1):15–26. 10.2307/3546992

[35] FAO: CGIAR Research Priorities for Marginal Lands.2000 Reference Source

[36] FAO: The state of the world’s land and water resources for food and agriculture.2011 Reference Source

[37] Nijsen M Smeets E Stehfest E: An evaluation of the global potential of bioenergy production on degraded lands. *GCB Bioenergy.* 2012;4(2):130–47. 10.1111/j.1757-1707.2011.01121.x

[38] Gelfand I Sahajpal R Zhang X: Sustainable bioenergy production from marginal lands in the US Midwest. *Nature.* 2013;493(7433):514–7. 10.1038/nature11811 23334409

[39] Carrete M Tella JL: Is assisted colonization feasible? Lessons from past introductions. *Front Ecol Environ.* 2012;10(1):12–3. 10.1890/12.WB.004

[40] McDonald-Madden E Runge MC Possingham HP: Optimal timing for managed relocation of species faced with climate change. *Nat Clim Chang.* 2011;1(5):261–5. 10.1038/nclimate1170

[41] Watson JE Iwamura T Butt N: Mapping vulnerability and conservation adaptation strategies under climate change. *Nat Clim Chang.* 2013;3(11):989–94. 10.1038/nclimate2007

[42] Iwamura T Wilson KA Venter O: A climatic stability approach to prioritizing global conservation investments. *PLoS One.* 2010;5(11):e15103. 10.1371/journal.pone.0015103 21152095 PMC2994894

[43] Sherwood SC Bony S Dufresne JL: Spread in model climate sensitivity traced to atmospheric convective mixing. *Nature.* 2014;505(7481):37–42. 10.1038/nature12829 24380952

[44] Ellison AM: Political borders should not hamper wildlife. *Nature.* 2014;508(7494):9. 10.1038/508009a 24695280

[45] Harsch MA Hulme PE McGlone MS: Are treelines advancing? A global meta-analysis of treeline response to climate warming. *Ecol Lett.* 2009;12(10):1040–9. 10.1111/j.1461-0248.2009.01355.x 19682007

[46] Hitch AT Leberg PL: Breeding distributions of north American bird species moving north as a result of climate change. *Conserv Biol.* 2007;21(2):534–9. 10.1111/j.1523-1739.2006.00609.x 17391203

[47] Ryan L: Translocation and Ancient Woodland.Grantham;2013.

[48] Lindo Z Winchester N: Out on a limb: microarthropod and microclimate variation in coastal temperate rainforest canopies. Didham R, Basset Y, editors. *Insect Conserv Divers* 2013;6(4):513–21. 10.1111/icad.12010

[49] Keppel G Van Niel KP Wardell-Johnson GW: Refugia: identifying and understanding safe havens for biodiversity under climate change. *Glob Ecol Biogeogr.* 2012;21(4):393–404. 10.1111/j.1466-8238.2011.00686.x

[50] Pearson RG Dawson TP: Long-distance plant dispersal and habitat fragmentation: identifying conservation targets for spatial landscape planning under climate change. *Biol Conserv.* 2005;123(3):389–401. 10.1016/j.biocon.2004.12.006

[51] Mackey BG Watson JE Hope G: Climate change, biodiversity conservation, and the role of protected areas: An Australian perspective. *Biodiversity.* 2008;9(3–4):11–8. 10.1080/14888386.2008.9712902

[52] Seddon PJ Moehrenschlager A Ewen J: Reintroducing resurrected species: selecting DeExtinction candidates. *Trends Ecol Evol.* 2014;29(3):140–7. 10.1016/j.tree.2014.01.007 24513302

[53] Hiss T: Can the World Really Set Aside Half of the Planet for Wildlife? *Smithsonian Magazine.* 2014 Reference Source

[54] Dirzo R Young HS Galetti M: Defaunation in the Anthropocene. *Science.* 2014;345(6195):401–6. 10.1126/science.1251817 25061202

[55] Iwamura T Guisan A Wilson KA: How robust are global conservation priorities to climate change? *Glob Environ Chang.* 2013;23(5):1277–84. 10.1016/j.gloenvcha.2013.07.016

[56] Mittermeier RA Turner WR Larsen FW: Global biodiversity conservation: the critical role of hotspots.In: Zachos FE, Habel JC, editors. *Hotspots Revisited: Earth’s Biologically Richest and Most Endangered Terrestrial Ecoregions* Berlin, Heidelberg: Springer Berlin Heidelberg,2011;3–8. 10.1007/978-3-642-20992-5_1

[57] Oldeman LR Hakkeling RTA Sombroek WG: World map of the status of Human-induced soil degradation. An Explanatory Note.1990 Reference Source

