# Supplementary material for: Can ecosystem-scale translocations mitigate the impact of climate change on terrestrial biodiversity? Promises, pitfalls, and possibilities: Ecosystem-scale translocations
Source: F1000Res. 2016 Feb 8;5:146. [Version 1] doi: 10.12688/f1000research.7914.1 (PMC4784018; doi:10.12688/f1000research.7914.1)
Supplement: Peer review history

# Peer review: Referee response for version 1

**DOI:** 10.5256/f1000research.8519.r12537

**Published:** 2016-03-07

**Authors:** John Box

**Affiliations:** 1 Atkins Ltd, Birmingham, UK

This is an extremely exciting but very challenging topic which certainly needs to be raised in order to stimulate the further experimental field trials that are required. Critical success/failure parameters need to be established across the wide range of habitats and communities that could be involved.

I have read this submission. I believe that I have an appropriate level of expertise to confirm that it is of an acceptable scientific standard.

# Peer review: Referee response for version 1

**DOI:** 10.5256/f1000research.8519.r12433

**Published:** 2016-02-15

**Authors:** Michael J Samways

**Affiliations:** 1 Department of Conservation Ecology and Entomology, Stellenbosch University, Stellenbosch, South Africa

This is an exciting paper which is highly contemporary and challenging to biodiversity conservation in the future.  I personally have huge ethical issues with this EST approach as it completely violates sense of place, and also the actual physical aspects are highly embedded in chaos theory, with the chances of predicting outcomes very slim, including the great risks to indigenous biota. Furthermore, even the baselines are changing: with receptor sites under the same stresses as the donor sites, despite the (highly speculative) maps provided here in the MS. However, as futurists we must keep all options open, as the next few decades are going to be very uncertain, and we may have to consider all sorts of strategies which may seem outrageous to us now. While I don’t agree with this EST approach, the authors present some very interesting “promises, pitfalls and possibilities” which although highly contentious are well articulated…and this is the strength of the MS. It is a wonderful trigger for thought and discussion. In short, I recommend the MS be indexed.

I have read this submission. I believe that I have an appropriate level of expertise to confirm that it is of an acceptable scientific standard.
